# A Primary Testicular Diffuse Large B-cell Lymphoma Belonging to the Germinal Center B-cell-like Group

**DOI:** 10.4021/jocmr2009.12.1284

**Published:** 2010-02-05

**Authors:** Mona Mlika, Ines Chelly, Mohamed Benrhouma, Slim Haouet, Ali Horchani, Mohamed Moncef Zitouna, Nidham Kchir

**Affiliations:** a Department of Pathology, La Rabta Hospital, Tunisia; b Department of Urology, La Rabta Hospital, Tunisia

## Abstract

Testicular lymphoma was first reported by Malassez and Curling in 1866. Primary testicular lymphoma constitutes only 1 - 7% of all testicular neoplasms and less than 1% of all non Hodgkin lymphoma. The authors report a new case of primary testicular lymphoma and highlight its diagnostic and therapeutic challenge. We report the case of a 26-year old man without a particular past medical history, who presented with a painful right testicular swelling that he has noticed for several weeks. Radiological findings consisted in multiple hypoechoic masses that corresponded in histological examination to a diffuse intratubular lymphomatous infiltration situated away from the spermatic cord, the epididymis, ductuli efferentes and rete testis. Immunohistochemical study showed positivity for leukocytic common antigen (CD45), B-cell marker (CD20) and bcl 6. The patient underwent full staging for lymphoma showing no evidence of extra-testicular involvement by lymphoma and no lymph nodes. The diagnosis of stage I primary testicular large B-cell lymphoma of germinal center B-cell-like group was made. The patient is now treated by chemotherapy. Primary testicular lymphoma is a rare tumour whose diagnosis is based on histological findings. There are non consensual etiological or predisposing factors. Treatment modalities consist in surgical excision, chemotherapy and radiation therapy but the accurate procedures are not standardized. Factors that have been linked to more favorable outcomes include younger patient age, localized disease, presence of sclerosis at pathologic analysis, smaller tumour size, lower histological tumor grade and lack of epididymal or spermatic cord involvement.

**Keywords:**

Testicular lymphoma; Germinal center; B-cell

## Introduction

Primary testicular lymphoma is a rare tumor accounting for 1% of all testicular non Hodgkin lymphoma [1]. It is defined by the primary localization of the tumour in the testis at presentation. The authors report a new case of primary testicular lymphoma and highlight its diagnostic and therapeutic challenge.

## Case Report

**Figure 1:**
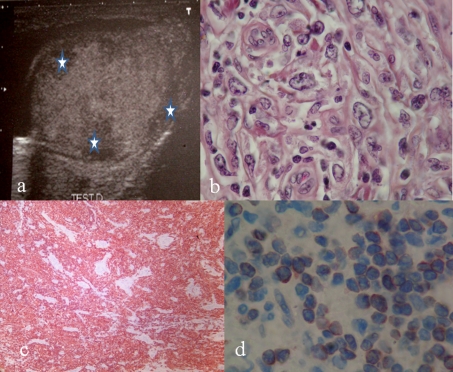
a: Ultra-sound examination showed an enlarged, heterogeneous testis with multiple hypoechoic masses (star); b: Malignant cells with large and scant cytoplasm and large vesicular nuclei (HE x 400); c: Positivity of the tumor cells with the B cell marker (HE x 400); d: Positivity of tumor cells with bcl 6 (HE x 400).

We report the case of a 26-year old man without a particular past medical history, who presented with a painful right testicular swelling that he has noticed for several weeks. There was no reported history of trauma, night sweets, fever or chills. Scrotal examination revealed a firm and enlarged testis with a homolateral hydrocele. The remainder of the clinical examination was noncontributory. The ultra-sound examination showed an enlarged, heterogeneous testis with multiple hypoechoic masses (Fig. 1a). Laboratory tests, especially the serum lactate dehydrogenase (LDH), the serum alpha-fetoprotein (αFP) and serum beta human chorionic gonadotropin (βHCG) levels were normal. An excision of the right testis was performed. It measured 6 x 5 x 3 cm and had an attached spermatic cord of 6 cm. The testicular section revealed 4 intra-testicular masses measuring 3, 4, 3 and 5 mm. they were distant from the epididymis and the spermatic cord. The light microscopy demonstrated a diffuse intratubular lymphomatous infiltration situated away from the spermatic cord, the epididymis, ductuli efferentes and rete testis (Fig. 1b). The malignant cells were large with scant cytoplasm and large vesicular nuclei. The paraffin immunohistochemical staining showed positivity for leukocytic common antigen (CD45), B-cell marker (CD20) and bcl 6 (Fig. 1c, d). Tumour cells did not express CD10 and bcl 2 antigens. The patient underwent full staging for lymphoma including tomography of the chest, abdomen and pelvis, positron emission tomography, and bone marrow biopsy. None of which revealed any evidence of extra-testicular involvement by lymphoma or any lymph nodes. The diagnosis of stage I primary testicular large B-cell lymphoma of germinal center-B-cell like group was made. The patient is now treated by chemotherapy.

## Discussion

Testicular lymphoma was first reported by Malassez and Curling in 1866 [2,3]. Primary testicular lymphoma constitutes only 1 - 7% of all testicular neoplasms and less than 1% of all non Hodgkin lymphoma [4]. The mean age at presentation is 60 years, but the recent published cases concerned patients younger than the past reported series and considered that this fact has a positive effect on the outcome of the patients [1,5]. According to the recent publications, our patient is young and is aged only 26 years. The typical presentation is a testicular painless mass of variable size that is usually unilateral [6]. However, at presentation, a bilateral involvement is noticed in up to 10% of the cases [6]. This fact made many authors suppose the possible multicenter origin since there is no direct lymphatic or venous connection between the right and left testis, but the fact that there are patients, like our patient, who have had localized disease and have been cured through orchidectomie alone favors the existence of testicular non Hodgkin lymphoma as primary disease [4]. Primary testicular lymphoma has tendency to spread to several extra-nodal sites including the central nervous system (CNS), skin, lung, pleura, waldeyers ring, soft tissue and eyes [1,7]. The imaging features reflect its infiltrative but nondestructive characteristics. At ultra-sound examination, the normal homogeneous echogenic testis is replaced focally or diffusely with hypoechoic vascular lymphomatous tissue [8]. LDH levels have been correlated with tumour aggressiveness, whereas other tumour markers such as βHCG and αFP are rarely elevated in TNHL cases [4]. In our case, the LDH, βHCG and αFP levels were normal. Histological examination is the only means of diagnosis. It can be made on biopsy or surgical specimen. Testicular lymphoma is locally aggressive and can typically infiltrate the epididymis, spermatic cord or scrotal skin [9]. The predominant histology is diffuse large B-cell lymphoma (DLBCL). It is reported in more than 70% of the cases [6]. The other sub-types include follicular lymphoma, plasmacytoma, lymphoblastic and Burkitts like lymphoma. The DLBCL is classified as germinal center B-cell-like or non germinal center B-cell-like by means of immunohistochemical expression of CD10, bcl 6 and MUM1 [10]. The non-germinal center B-cell-like sub-group is the most frequent; it exhibits a high proliferative activity [4]. On the other hand, the germinal center B-cell type, like our reported case, is seen mostly in HIV-positive patients and has a better overall survival [6].

Histopathological differentiation of testicular lymphomas from germinal tumors is usually a challenge but these lymphomas generally appear more lobulated with well defined borders at ultra-sound examination [9]. Other conditions might mimic testicular lymphoma such as granulomatous orchitis, pseudolymphoma, palsmacytoma and rhabdomyosarcoma [4]. There are non consensual etiological or predisposing factors. Various reports have implicated prior trauma, chronic orchitis, cryptorchidism and filariasis of the spermatic cord as risk factors [9]. Treatment modalities consist in surgical excision, chemotherapy and radiation therapy, but the accurate procedures are not standardized. Early retrospective studies indicated that local treatment with surgery alone or surgery plus radiotherapy and chemotherapy without antracycline provides suboptimal disease control even in localized disease. Recently, combined modality treatment with systemic doxorubicine-based chemotherapy, prophylactic intrathecal chemotherapy and scrotal radiotherapy has been recommended because of the relapse risk at extra-nodal sites such as the CNS and controlateral testis. Despite these more aggressive treatment modalities, prognosis is often poor, even in the localized disease with the two-year relapse rate exceeding 50%. Factors that have been linked to more favorable outcomes include younger patient age, localized disease, presence of sclerosis at pathologic analysis, smaller tumour size, lower histological tumour grade and lack of epididymal or spermatic cord involvement [9]. According to these prognostic factors, our patient seems to have a good outcome because of his young age, the localized disease and the germinal center B-cell-like type tumour.
